# Hyperperfusion in the cerebellum lobule VIIb in patients with epileptic seizures

**DOI:** 10.1186/s12883-022-02882-0

**Published:** 2022-09-16

**Authors:** Kazuaki Sato, Kazuki Nakahara, Kaoru Obata, Ryota Matsunari, Rie Suzuki-Tsuburaya, Hiromitsu Tabata, Masako Kinoshita

**Affiliations:** 1 Department of Neurology, National Hospital Organization Utano National Hospital, 8 Ondoyama-Cho, Narutaki, Ukyoku, Kyoto, 616-8255 Japan; 2 Department of Radiology, National Hospital Organization Osaka National Hospital, Osaka, Japan; 3 Department of Neurology, Kurashiki Central Hospital, Kurashiki, Okayama, Japan; 4 Department of Forensics Medicine, Kyoto Prefectural University of Medicine, Kyoto, Japan; 5 Department of Pediatric Neurology, National Hospital Organization Utano National Hospital, Kyoto, Japan; 6 Department of Neurology, Osaka Kaisei Hospital, Osaka, Japan

**Keywords:** Epilepsy, Cerebellum, Lobule VIIb, Magnetic resonance image, Arterial spin labeling, Hyperperfusion

## Abstract

**Background:**

The cerebellum plays an important role in motor control, however, its involvement in epilepsy has not been fully understood. Arterial spin labelling perfusion magnetic resonance image (ASL) is a noninvasive method to evaluate cerebral and cerebellar blood flow. We investigated cerebellar perfusion in patients with epileptic seizures using ASL.

**Methods:**

Adult patients with epileptic seizures who underwent ASL in three post labeling delay (PLD) conditions (1525, 1800, and 2500 msec) and conventional electroencephalography (EEG) on the same day were investigated. Clinical and EEG characteristics of them were retrospectively analyzed.

**Results:**

Six patients (6 women, age; 36.2 ± 17.9 years (mean ± SD)) showed hyperperfusion in selective areas in the cerebellar paravermis of lobule VIIb. One patient with generalized epilepsy (tentative diagnosis of juvenile myoclonic epilepsy or epilepsy with myoclonic absences) showed unilateral hypoperfusion in PLD 1525 msec and hyperperfusion in PLD 1800 and 2500 msec at the area while EEG showed generalized spike-wave complexes. After successful treatment, these perfusion abnormalities disappeared. In two patients with focal epilepsy manifesting with asymmetrical motor symptoms, cerebellar hyperperfusion was found on the opposite side to the seizure focus estimated by seizure semiology. Besides hyperperfusion of the VIIb lobule, hypoperfusion at the same area was detected in shorter PLD condition in four patients and in longer PLD condition in one patient.

**Conclusion:**

The cerebellar paravermis of lobule VIIb can be a component of motor circuit and participate in epileptic network in humans. Cerebellar perfusion abnormalities can be associated with neurovascular coupling via capillary bed.

## Background

The cerebellum plays an important role in motor control. The cerebello-thalamo-cortical loop modulates the cerebro-basal ganglia loop. Cerebellar ataxia is one of the four cardinal features of progressive myoclonus epilepsy. Pathological involvement of cerebellum has been demonstrated in juvenile myoclonus epilepsy (JME) [1], benign adult familial myoclonic epilepsy (BAFME) [2], juvenile absence epilepsy [3], and nonconvulsive partial status epileptics [4]. Furthermore, cerebellar hemisphere is one of the target sites of neuromodulation using electric stimulation in intractable epilepsy. Cerebellar stimulation was reported to decrease seizure activity in animal models of epilepsy [5]. In patients with intractable epilepsy, previous reports showed effectiveness of cerebellar stimulation to seizure suppression, while the effectiveness and procedures of cerebellar stimulation are still controversial [6]. To elucidate the mechanism of influence of the cerebellum on the cerebral cortex is very important in further development of treatment strategies in patients with epilepsy.

Arterial spin labeling (ASL) is a noninvasive method to evaluate cerebral blood flow (CBF) using magnetically-labeled water in the blood as an endogenous tracer. Hyperperfusion of ASL with post labeling delay (PLD) of approximately 1500 msec more sensitively correlates with epileptic seizures than high intensity of diffusion weighted images and hypoperfusion with PLD of 1500 msec is seen during interictal period [4, 7]. Thus far, little is known about involvement of cerebellum to pathology of epilepsy.

Our objective is to investigate cerebellar perfusion in patients with epileptic seizures using arterial spin labeling perfusion magnetic resonance image. Part of this manuscript was presented in the American Academy of Neurology Annual Meeting 2020 in an abstract form [8].

## Methods

In this case-series study, we retrospectively checked medical record of patients who visited the Adult Epilepsy Clinic in the National Hospital Organization Utano National Hospital between August 2017 and May 2018, and enrolled patients who fulfilled inclusion criteria; 1) adult patients with epileptic seizures, 2) underwent ASL in three PLD conditions (1525, 1800, and 2500 msec) (Ingenia 3.0 T CX, Philips), 3) anatomical images using fluid-attenuated inversion recovery, diffusion weighted imaging, or susceptibility weighted imaging sequences were obtained in combination with ASL images, 4) conventional electroencephalography (EEG) (EEG1214, Nihon Kohden) was recorded on the same day, and 5) whose ASL images showed spotty hyperperfusion in the cerebellar hemisphere. Clinical and EEG characteristics of them were retrospectively analyzed. MRI scan took approximately 30 min, and EEG was recorded for approximately 30 min. In patients treated with antiepileptic drugs, regular medications were taken as usual.

The recordings were performed as a part of an intensive clinical evaluation. On the basis of the noninvasive case-accumulation study design with assured anonymity, the current study was exempt from the need for the institutional ethics committee approval and informed consent.

## Results

### Patient demographics

**Fig. 1:**
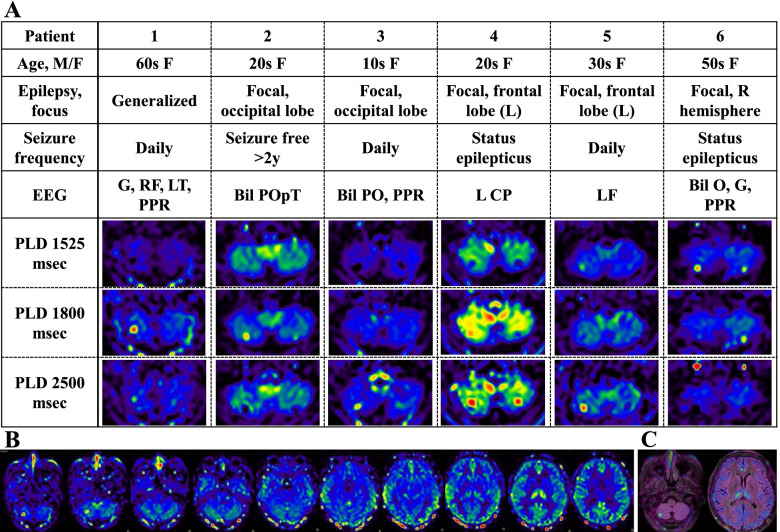
**A** Six patients with epilepsy, showing hyperperfusion of ASL in selective areas the cerebellar paravermis of lobule VIIb. Four patients (Patient 1, 3, 4, and 5) showed hypoperfusion of the VIIb lobule in short PLD conditions (1525 msec in Patient 1 and Patient 4, 1525 and 1800 msec in Patient 3, and 1800 msec in Patient 5) and showed hyperperfusion of the same region in longer PLD conditions. In Patient 4 and Patient 5 with focal epilepsy manifesting with asymmetrical motor symptoms, cerebellar hyperperfusion was found on the opposite side to the seizure focus estimated by seizure semiology. **B** Axial ASL images of Patient 1. At the bottom of the right cerebellum, hypoperfusion in PLD 1525 msec and hyperperfusion in PLD 1800 and 2500 msec are seen. In the left thalamus, hypoperfusion in PLD 1800 and 2500 msec is shown. **C** Co-registration of ASL to FLAIR images. ASL: arterial spin labeling, PLD: post labeling delay, FLAIR: fluid-attenuated inversion-recovery, M/F: male/female, EEG: electroencephalogram, R: right, L: left, Bil: bilateral, G: generalized, F: frontal, C: central, P: parietal, O: occipital, T: temporal, pT: posteriotemporal, PPR: photoparoxysmal response

Six patients (6 women, age; 36.2 ± 17.9 years (mean ± SD)) fulfilled the inclusion criteria (Fig. 1, Table 1). One of six patients (Patient 1) was diagnosed as generalized epilepsy, and the rest were diagnosed as focal epilepsy. Patient 4 developed status epilepticus and was treated with antiepileptic drug injection from 3 hours to 1 hour before MRI examination. Patient 1 exhibited frequent myoclonic seizures of the eyelids and hands and Patient 3 showed focal aware seizures of dizziness during EEG. No patient showed cerebellar ataxia by thorough neurological examination including extraocular movement, speech, limb coordination, and gait in interictal period. Patient1 and Patient 3 were not treated with antiepileptic drugs. Order of examinations were EEG to MRI in 2 patients and opposite in the rest, and interval between examinations ranged from 24 to 200 min.

**Table 1:** Patient demopgraphics

Patient	Age, M/F	Antiepileptic medication	Order of examinations	Interval between examinations (min)	Seizures during and between examinations	Time since last seizure
1	60s F	none	EEG - > MRI	59	(+), frequent eyelid myoclonia and hand/fingers myoclonic seizures	–
2	20s F	LTG 25 mg	MRI - > EEG	35	(−)	> 2 years
3	10s F	none	EEG - > MRI	200	(+), FAS (dizziness) during EEG	–
4	20s F	VPA 600 mg, LTG 350 mg, LCM 450 mg; DZP 20 mg (i.m. and i.v.) and fosPHT 1125 mg (d.i.v.) from 180 min to 60 min before MRI	MRI - > EEG	34	(+), status epilepticus of FIAS (conjugation to R, tonic convulsion of R limbs)	–
5	30s F	PHT 275 mg	EEG - > MRI	24	(−)	1 day
6	50s F	LEV 1000 mg	EEG - > MRI	37	(−)	2 years

*M/F* male/female, *EEG* electroencephalogram, *MRI* magnetic resonance image, *R* right, *FAS* focal aware seizures, *FIAS* focal impaired awareness seizures, *LTG* lamotrigine, *VPA* valproate, *LCM* lacosamide, *DZP* diazepam, *fosPHT* fosphenytoin, *i.m*. intramuscular, *i.v.* intravenous, *d.i.v* intravenous drip

### Case presentation

#### Patient 1

**Fig. 2:**
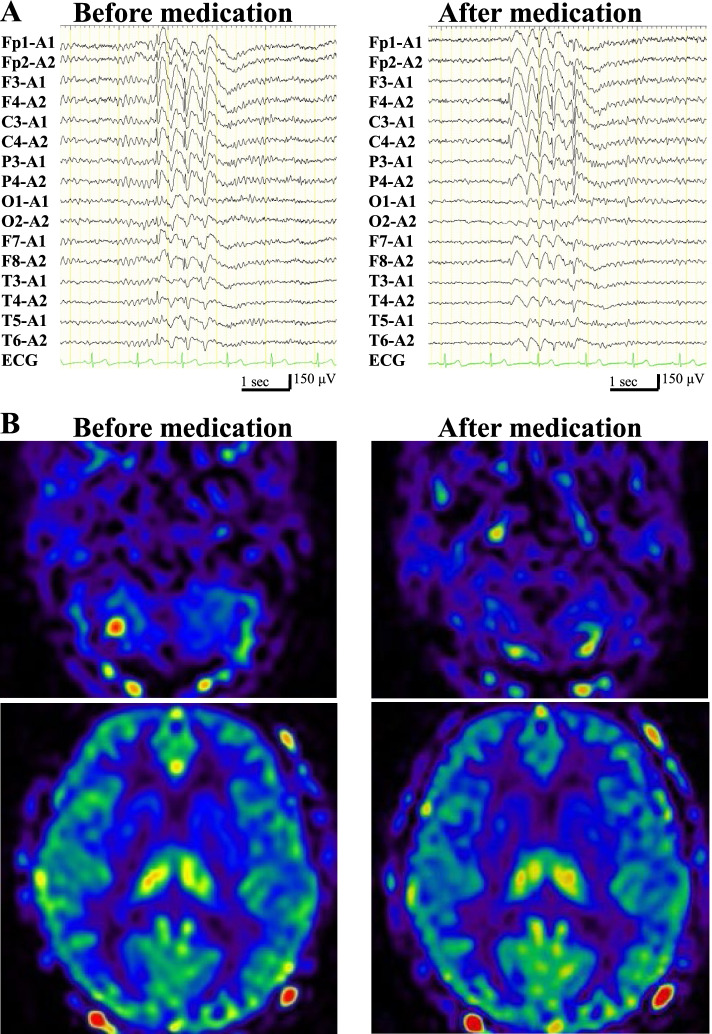
**A** Electroencephalogram of Patient 1. Before treatment (left), generalized spike-wave complexes are seen; focal spikes in the right frontal and left temporal areas and photoparoxysmal responses were noticed (data not shown). After treatment (right), amplitude of spikes is decreased. Electrodes placement: International 10–20 + T1/T2. Sampling rate: 500 Hz. AC amplifier low frequency filter: 0.08 Hz. **B** Arterial spin labeling images of Patient 1, post labelling delay of 1800 msec. Before treatment (left), the right cerebellar hyperperfusion and the left thalamic hypoperfusion are shown. After treatment (right), these abnormalities are not detected

Initial representative case was a right-handed female in her 60s, who presented with myoclonus of hands and face, myoclonic seizures, and generalized tonic-clonic seizures. She had a past history of surgical resection of benign pancreatic tumor. Family history was unremarkable. At the evaluation before treatment, unilateral hypoperfusion in PLD 1525 msec and hyperperfusion in PLD 1800 and 2500 msec in the right lobule VIIb, and hypoperfusion in PLD 1800 msec and 2500 msec in the left thalamus were shown, while EEG showed generalized spike-wave complexes (SWC), focal spikes in the right frontal and left temporal areas, and photoparoxysmal responses (Fig. 2). She was diagnosed with generalized epilepsy (myoclonic absence epilepsy or juvenile myoclonus epilepsy). Valproate was not effective. She underwent serial evaluation by both ASL and EEG after commencement of treatment with an anti-epileptic drug. After successful treatment by clonazepam, these perfusion abnormalities disappeared while EEG showed decreased amplitude of generalized SWC.

#### Patient 2

A teenage female presented with focal impaired awareness seizures preceded by visual hallucination of flickering lights in hemifield. She started having loss of consciousness attacks at age 6. At age 13 she developed paroxysmal dyskinesia which impaired writing, walking, or playing trumpet, and was diagnosed with paroxysmal kinesigenic dyskinesia. However, she had frequent visual symptoms and headache and in the previous year she showed convulsive seizures with consciousness disturbance. EEG showed frequent rhythmic slow activities. Treatment with sufficient dose of carbamazepine suppressed her seizures for more than 2 years. Considering possible chance of pregnancy and teratogenicity of carbamazepine, the medication was switched to lamotrigine 25 mg/day 1 month before examination.

#### Patient 3

A teenage female was referred to our epileptic clinic for evaluation of monthly attacks of severe dizziness. She developed her first seizure with dizziness at age 12 and the symptoms progressed to show loss of body sensation, eyelid twitch, impairment of voluntary movement, and dyspnea. She had never tried antiepileptic medication. During EEG she had her typical focal aware seizure with dizziness, and EEG correlate was determined. Combination therapy with multiple antiepileptic drugs is effective to her seizures.

#### Patient 4

She showed mental retardation and educated in special class for handicapped children since elementary school. In her late teens she developed loss of consciousness attacks but these symptoms were considered as pseudoseizures in previous hospitals due to inappropriate behavior. Her focal impaired awareness seizures comprised of loss of sensation and tonic convulsion of the right limbs, nausea, vomiting, and consciousness disturbance with conjugated gaze and dilated pupils. She developed monthly status epilepticus and referred to our clinic in her 20s. Combination therapy with valproate, lamotrigine, and lacosamide was partially effective. On the day of regular clinic visit in her mid 20s, she developed status epilepticus and was treated with diazepam and fosphenytoin injection from 3 hours to 1 hour before MRI examination. ASL showed diffuse hyperperfusion especially in the left primary somatosensory area besides cerebellar spotty lesion, and EEG showed spikes and EEG seizure pattern maximum in posterior-temporal area. After each attack of status epilepticus, residual right rigospastic paraparesis and dysdiadochokinesis lasted for several weeks. Underlying etiologies including autoimmune encephalitis were considered but she refused intensive evaluation.

#### Patient 5

She developed generalized convulsion in her 20s. Phenytoin was effective but she often had seizures when she missed dose, and daily nocturnal seizures of the right arm. Lamotrigine add-on caused skin rush. One week before examination, she had loss of consciousness and fall at office during the day. Neurological examination showed positive Barre sign on the right arm and dysdiadochokinesis of left-right alternating hand movement.

#### Patient 6

A female in her 50s developed status epilepticus starting with head and eye version to left subsequent to viral enteritis and was treated in previous hospitals. Afterwards she was seizure free with levetiracetam 1000 mg/day for 2 years, thus stopped medication 3 months before examination. EEG showed generalized and bioccipital spike-wave-complexes and photo-paroxysmal responses. ASL and EEG showed abnormality but she refused to re-start medication.

#### ASL findings

ASL images of all patients showed hyperperfusion in selective areas the cerebellar paravermis of lobule VIIb.

In two patients with focal epilepsy manifesting with asymmetrical motor symptoms (Patient 4 and Patient 5), cerebellar hyperperfusion was found on the opposite side to the seizure focus estimated by seizure semiology (Fig. 1).

Four patients (Patient 1, 3, 4, and 5) showed hypoperfusion of the VIIb lobule in short PLD conditions (1525 msec in Patient 1 and Patient 4, 1525 and 1800 msec in Patient 3, and 1800 msec in Patient 5) and showed hyperperfusion of the same region in longer PLD conditions (Fig. 1). Patient 2 showed hyperperfusin in PLD 1800 msec and hypoperfusion in PLD 2500 msec at the same area.

## Discussion

The present study evaluated cerebellar perfusion by using ASL in three PLD conditions in adult patients with epilepsy. To the best of our knowledge, this is the first study to demonstrate hyperperfusion in the cerebellar paravermis of lobule VIIb. Thus, the cerebello-thalamo circuit can participate in epileptic functional network in human epilepsy.

Previous reports have shown the relationship between several epilepsy syndromes and the cerebellum. Studies using functional MRI found an enhanced functional connectivity between the left cerebellar lobule VIIb and the right frontal pole in BAFME [2] and decreased volume of the cerebellum in JME patients [1]. Cerebellar hemispheres mainly consist of VI–X lobules, and the VIIb lobules are at the bottom. Each of VII–VIII lobules modulates voluntary movement of hands, e.g., reaction to force or to visual stimuli [9]. Lobules VII are activated during tasks that demands on cognitive functions including language, working memory, and executive function [10]. However it is unlikely that the hyperperfusion of lobules VIIB in this study is physiological phenomenon because was demonstrated during MRI imaging at rest.

Efferent projections originate from the cerebrum synapse to the pontine nuclei and then primarily to the contralateral cerebellar cortex. Purkinje cell axons conduct the entire output of the cerebellar cortex, projecting to the deep cerebellar nuclei (e.g., dentate nucleus, interposed nucleus) in the underlying white matter, where the gamma-aminobutylic acid released by their terminals has an inhibitory effect. The cerebral cortex receives excitatory synapse from cerebellar nuclei via ventrolateral nucleus of the thalamus [11]. In mouse model of absence epilepsy, group discharges of Purkinje cells are recorded during generalized rhythmic spike discharges [12]. Hyperperfusion of the right lobule VIIB and hypoperfusion of the opposite side of the thalamus in Patient 1 before medication can be the result of activation of Purkinje cells caused by epileptic discharge of the cerebrum, which then inhibits the thalamic activities. After medication, along with decrease of SWC, these phenomena disappeared.

In patients with focal epilepsy, side of hyperperfusion was related to clinical presentation. Patient 4 and Patient 5, whose seizure focus resides in the left frontal lobe manifesting with asymmetrical motor symptoms, showed hyperperfusion of right lobules VIIb. Patient 4, who was in status epilepticus during the examinations, showed hyperperfusion of both cerebellar hemispheres in concordant with a previous report on nonconvulsive status epilepticus [4], however, remarkably significant hyperperfusion of VIIb can suggest marked involvement of motor circuit of hand movement. In addition, inflammatory etiology such as autoimmune encephalitis can exaggerate excitability of motor network. Patient 6 showed cerebellar hyperperfusion ipsilateral to the seizure focus presumed by the initial seizure semiology, but EEG with generalized epileptiform discharges suggests involvement of both hemispheres. Visual motor integration can also be important because seizures of Patient 2 and Patient 3 originate from occipital lobe and photoparoxysmal responses were noticed in Patient 1, Patient 3, and Patient 6.

ASL reflects CBF, and quantitative measurement of CBF by ASL depends on arterial transit time which is the time delay between the labeled blood in the cervical arteries and the arrival of the labeled blood in cerebral tissue. Therefore, the setting of PLD is very important to evaluate CBF correctly. In patients with chronic occlusive cerebrovascular disease, PLD 1500 msec underestimates CBF of affected side whereas PLD 2500 msec can evaluate hemodynamic state precisely [13]. Neuronal activation also causes changes in cerebral microcirculatory. Microcirculatory response to functional stimulation leads to a pronounced decrease in vascular transit time and a dilatation of the capillary bed [14]: mean transit time in cerebral capillaries drops from 1.34 ± 0.4 s at rest to 0.78 ± 0.02 s during activation. Epileptic seizure originates from firing of neuron, and thus resultant microcirculatory change may occur.

We evaluated CBF of the brain with three PLD conditions (1525, 1800, and 2500 msec). In addition to the hyperperfusion of the VIIb lobule, hypoperfusion at the same area was detected in shorter PLD condition in four patients and in longer PLD condition in one patient. Hypoperfusion may reflect the decrease of the vascular transit time and hyperperfusion may reflect a dilatation of the capillary bed evoked by neural activation. The phenomena can be associated with characteristics of the cerebellar vasculature which is mainly comprised of the capillary net.

A decrease of metabolism and blood flow in the cerebellar hemisphere contralateral to a supratentorial infarct is called as crossed cerebellar diaschisis (CCD). CCD has been evaluated using single-photon emission computed tomography (SPECT) or positron-emission tomography whose special resolution is relatively low. A recent study evaluated CBF derived from ASL and showed that was equivalent to CBF derived from SPECT, and CCD was shown in diffusely in cerebellar hemisphere [15]. In contrast, hyperperfusion and hypoperfusion of our patients were detected in restricted region of the cerebellar paravermis of lobule VIIb, suggesting a totally different mechanism from CCD.

## Conclusions

The cerebellar paravermis of lobule VIIb can be a component of motor circuit and participate in epileptic functional network in humans. Further studies to correlate hyperperfusion of the VIIb to precise motor manifestation, etiology, and prognosis, and to explore how to modulate motor circuit, may reveal utility of the phenomenon in clinical practice of patients with epilepsy. Accumulation of ASL data with several PLD conditions is warranted to elucidate functional network of the brain, neurovascular coupling, and treatment strategy for intractable epilepsy.

## Data Availability

The data that support the findings of this study are available on request from the corresponding author [M.K.]. The data are not publicly available due to information that could compromise research participant privacy.

## Abbreviations

JME: Juvenile myoclonus epilepsy
BAFME: Benign adult familial myoclonic epilepsy
ASL: Arterial spin labeling
CBF: Cerebral blood flow
PLD: Post labeling delay
EEG: Electroencephalography
SWC: Spike-wave complexes
CCD: Crossed cerebellar diaschisis
SPECT: Single-photon emission computed tomography
